# Agreement between ELISA and plaque reduction neutralisation assay in Detection of respiratory syncytial virus specific antibodies in a birth Cohort from Kilifi, coastal Kenya.

**DOI:** 10.12688/wellcomeopenres.15108.1

**Published:** 2019-02-18

**Authors:** Joyce U. Nyiro, Patience K. Kiyuka, Martin N. Mutunga, Charles J. Sande, Patrick K. Munywoki, J. Anthony G. Scott, D. James Nokes

**Affiliations:** 1 Epidemiology and Demography, Kenya Medical Research Institute-Wellcome Trust Research Programme, Centre for Geographic Medicine Research-Coast, Kilifi, Kenya; 2 Kenya Center for Disease Control and Prevention, Nairobi, Kenya; 3 Department of Infectious Disease Epidemiology, London School of Hygiene & Tropical Medicine, London, UK; 4 School of Life Sciences and Zeeman Institute (SBIDER), University of Warwick, Conventry, UK

**Keywords:** Respiratory syncytial virus, specific antibody, serological assays, agreement, birth cohort.

## Abstract

**Background: **Severe disease associated with respiratory syncytial virus (RSV) infection occurs predominantly among infants under 6 months of age. Vaccines for prevention are in clinical development. Assessment of the vaccine effectiveness in large epidemiological studies requires serological assays which are rapid, economical and standardised between laboratories. The objective of this study was to assess the agreement between two enzyme linked immunosorbent assays (ELISA) and the plaque reduction neutralisation test (PRNT) in quantifying RSV specific antibodies.

**Methods: **Archived sera from 99 participants of the Kilifi Birth Cohort (KBC) study (conducted 2002-2007) were screened for RSV antibodies using 3 methods: ELISA using crude RSV lysate as antigen, a commercial RSV immunoglobulin G (IgG) ELISA kit from IBL International GmbH, and PRNT. Pearson correlation, Bland-Altman plots and regression methods were used in analysis.

**Results:** There was high positive correlation between the IBL RSV IgG ELISA and PRNT antibodies (Pearson r=0.75), and moderate positive correlation between the crude RSV lysate IgG ELISA and PRNT antibodies (r= 0.61). Crude RSV lysate IgG ELISA showed a wider 95% limit of agreement (-1.866, 6.157) with PRNT compared to the IBL RSV IgG ELISA (1.392, 7.595). Mean PRNT titres were estimated within a width of 4.8 log _2_PRNT and 5.6 log _2_PRNT at 95% prediction interval by IBL RSV IgG and crude RSV lysate IgG ELISA, respectively.

**Conclusion: **Although, the IBL RSV IgG ELISA is observed to provide a reasonable correlate for PRNT assay in detecting RSV specific antibodies, it does not provide an accurate prediction for neutralizing antibody levels. An RSV neutralising antibody level is likely to fall within 2.4 fold higher and 2.4 fold lower than the true value if IBL RSV IgG ELISA is used to replace PRNT assay. The utility of an ELISA assay in vaccine studies should be assessed independent of the PRNT method.

## Introduction

Respiratory syncytial virus (RSV) is an important cause of annual epidemics of bronchiolitis and pneumonia in children less than five years of age worldwide ^1– 5^. The most severe disease occurs among infants under 6 months of age ^6^, making this group the principal target for RSV disease prevention.

To date there are no licensed vaccines for RSV. However, strategies to protect the young infant through immunisation are underway. One approach showing particular promise is to protect the infant over the first few months of life by maternal vaccination. A maternal RSV vaccine which is based on a post-fusion F protein nanoparticle design ^7^, is now undergoing phase 3 clinical trials ( NCT02624947). Standardised serological assays are needed if this or other vaccines show good efficacy as they go to effectiveness phase 3b/4 clinical trials.

The plaque reduction neutralisation test (PRNT) has been the preferred technique by most studies to determine the correlates of protective immunity to RSV ^8– 13^. This is because the assay determines the level of antibodies present that are presumed to be functional *in vivo* ^14^. For instance, a pseudovirion-based neutralisation assay was found to detect all neutralizing antibodies that directly arrest the virus and thus to provide protection against infection with Human papilloma virus ^15^. A study by Piedra *et al.* showed that serum antibody titres correlate with protection ^8^; a finding also supported by Stansbelle *et al*. who showed that neutralisation antibody titres were inversely associated with RSV hospitalization in infants <6months ^16^. Hence PRNT is considered the most appropriate serological assay for use in quantifying protective immunity correlates to RSV.

Although the PRNT is considered the “Gold standard” method for quantifying the level of RSV specific antibodies, it has several limitations: (i) PRNT antibody titres are currently not internationally standardised and therefore it is difficult to compare results across different studies, though work is under way to resolve this ^17^; (ii) the assay procedure is cumbersome - taking four to six days (according to method) to complete; and, (iii) the assay is costly in terms of laboratory technologist time. Hence, the PRNT is not ideal for large sero-epidemiological studies or large-scale vaccine trials.

Due to these limitations, it would be beneficial to adopt a serological assay which is quicker, cheaper and can be easily standardized, for use in large vaccine studies. An ELISA method is an option. However, the method measures epitopes of the target antigen and does not discriminate antibodies specific to neutralizing sites. Presently, there are two RSV specific IgG ELISA assays in our laboratory: an ELISA which uses crude RSV A2 culture lysate as antigen (Crude ELISA), and a commercial ELISA (IBL ELISA) which uses 96 well micro titre ELISA plates pre-coated with RSV F protein and RSV group A cell extract as antigen. We examined the agreement between ELISA assays and the PRNT in quantifying RSV specific antibodies using serum samples from a birth cohort in Kilifi, a coastal part of Kenya.

## Methods

### Study site and participants

This study was conducted in Kilifi, within the Kilifi Health and Demographic Surveillance System (KHDSS) in the coastal part of Kenya ^18– 20^. Archived serum and plasma samples were utilised arising from the Kilifi Birth Cohort (KBC) study conducted between 2002 and 2007, by the Kenya Medical Research Institute-Wellcome Trust Research Programme (KEMRI-WTRP). The KBC study was an observational study to investigate susceptibility to invasive pneumococcal disease among children. In this study, newborn participants numbering around 6000, recruited at the maternity ward or the child health clinic of Kilifi County Hospital (KCH), were followed for a 2-year period with blood samples collected at birth and then every 3 months. Samples were stored as plasma (for cord bloods) or serum (follow-up samples) at -80°C. Details of the birth cohort study are described elsewhere ^10, 18, 19^.

In the present study, a random sample set of 100 individuals from the KBC participants recruited from the KCH maternity ward, were selected from the study database. Description of the levels of maternal RSV specific neutralizing antibodies from blood samples of the 100 participants were provided in a previous publication ^10^.

### Ethical considerations

All parents and guardians gave written consent to have their children participate in the KBC study for storage of blood samples for use in future research. The use of the archived sample set was approved by the KEMRI-Scientific and Ethics Review Unit (SERU# 2307).

### Laboratory procedures

Archived plasma/serum samples from the KBC study which had been stored at -80°C were retrieved and screened using 3 serological assay techniques: (i) a PRNT (ii) an IgG ELISA assay using crude RSV lysate as antigen (crude ELISA) and (iii) an RSV IgG ELISA using a commercial ELISA kit (IBL ELISA).

***Plaque reduction neutralisation test (PRNT).*** The PRNT procedure for determining the titre of RSV neutralising antibodies has been described previously ^9^. The method incorporated a step in which serum samples were incubated at 56°C in a water bath for 30 minutes to inactivate complement cascade proteins. Each serum sample was repeatedly diluted 2-fold over ten consecutive dilutions and mixed with an equal volume of 50 plaque forming units (pfu) of RSV A2 virus (RSVA2 and Hep2 cells were a kind donation from Dr. Patricia Cane while she worked at the Health Protection Agency, UK). The virus-serum mixture (50µl per well) was dispensed over a confluent monolayer of Hep2 cells in a 96 well culture plate, incubated at 37°C for 1 hour and then underwent 4-hour cycles of rotation on an angled (about 30°) rotating platform (about 40 rev/minute) for 10 minutes and incubation in a 37°C CO _2_ incubator for 30 minutes. The plate was then incubated for 48 hours in a 37°C CO _2_ incubator. Fixation of cells was done by the addition of 100µl of fixation reagent (30% methanol+70% acetone). Plaques were detected by addition of a primary antibody (RSV F protein mouse monoclonal-BIO-RAD, Catalogue# MCA490) solution diluted 1:500 in PBS with 2 hours incubation at 37°C, followed by an addition of a 100µl/well of an anti-mouse HRP-conjugated secondary antibody (170-5047 Immun-Star Goat Anti-Mouse (GAM)- IgG (H/L) polyclonal antibody HRP–BIORAD) solution diluted 1:1000 in Phosphate Buffered Saline (PBS) with 1 hour incubation at room temperature. After each step, plates were washed manually three times using 200µl/well PBS buffer. Plaques were visualised by addition of 100µl/well detection reagent. This consisted of 16 µl of hydrogen peroxide and 0.6ml of 3-amino-9-ethlycarbazole 3.3mg/ml solution (20mg 3-amino-9-ethlycarbazole tablet were dissolved in 6.06ml of dimethyl sulphoxide (DMSO) to give a 3.3mg/ml solution) to 10ml of 20mM sodium acetate solution (pH 5.0-5.5). Reading and counting of the brown-stained RSV micro-plaques was done using an ELISpot reader (Autoimmun Diagnostika GmbH, Germany).

The dilution of a test serum sample required to induce 50% neutralization of a known titration of RSV A2 virus was determined using the Spearman Karber method ^9^. In addition, a panel of control samples from BEI Resources (BEI RSV Reference panel catalogue #NR-32832) was included in each batch of the PRNT assay to monitor reproducibility of the assay results and deterioration of the antibodies used. Results obtained from screening of the BEI samples were compared with PRNT values of the samples as previously tested in BEI resources laboratories.

***Crude RSV lysate IgG ELISA (Crude ELISA).*** Blood samples were tested for antibody concentration with an IgG based ELISA method using crude virus extract from lab-adapted RSV A2 culture following a local standard operating procedure ^21^. The crude virus RSV lysate preparation, optimal dilutions for RSV-A2 antigen, the serum dilutions and generation of a standard curve from a pooled adult serum were determined by a checkerboard titration as previously described ^21, 22^. In every run, one half of the 96 well plate (column 1-6) was coated with 50µl/well of RSV lysate (antigen), while the other half (column 7-12) was coated with 50µl/well of mock lysate (mock). The mock consisted of Hep2 cells without RSV virus prepared using same procedure as that of the RSV lysate. Plates were incubated overnight at 37°C, then blocked for 1 hour with 200µl/well of 5% skimmed milk at 37°C. Blocking buffer was flicked off. Diluted serum samples 100µl/well were dispensed to both the antigen and mock sides of the plate. The plates were washed 4 times with 200µl/well of 0.05% Tween 20 in PBS (PBS-T) using an ELISA plate washer. A secondary antibody [polyclonal antibody to human IgG heavy chains (Goat anti human IgG HRP antibody-KPL, Catalogue# 074-1002) (100µl/well) diluted 1:1000 in PBS buffer was added to each well and incubated for 1 hour at room temperature. The reaction was developed using 50µl/well of Ortho-Phenylenediamine dihydrochloride (OPD, Catalogue# P8412-100TAB, Sigma-Aldrich) solution as substrate (prepared just before use in the ratio 1mg of OPD in 1 ml of PBS and 1ul of hydrogen peroxide). The intensity of colour developed was read at 490nm using an ELISA reader (SYNERGY 4, BioTeK). All samples were run in duplicate to monitor reproducibility of results and variability arising from pipetting. Positive and negative controls were run on every plate and plotted on a graph over time to check for antigen deterioration of the standards or coating antigen. The OD values of the mock were subtracted from the OD values of the antigen lysate to give the final OD value of the serum sample. OD values of the standard serum dilutions were assigned arbitrary unit (AU) values. Serum samples were assigned arbitrary units of RSV specific IgG by comparison against a standard curve generated from the pooled adult serum (serum standard) tested in each ELISA plate.

***Commercial RSV IgG ELISA (IBL ELISA).*** Use of the commercial RSV IgG ELISA kit and the procedure followed manufacturer’s guidelines as described in the package insert (Cat#: RE56881- IBL International GmbH, D-22335 Hamburg, Germany). The ELISA procedure was based on a sandwich principle. In this assay, the 96 well plates were pre-coated with Vero E6 cells; strain long, F-protein; cell extract of RSV subgroup A (long strain) containing F-protein as well as G-protein as antigen (IBL communication). Specific antibodies from the serum samples binding to the pre-coated antigen were detected by a secondary antibody, rabbit anti-human conjugated to horse radish peroxidase enzyme. The reaction was developed using Tetramethylbenzidine(TMB) solution as substrate. The intensity of the colour developed, which is proportional to the concentration of IgG-specific antibodies detected, was read at 450nm using an ELISA reader (SYNERGY 4, BioTeK). The concentration of RSV IgG antibodies from the samples was estimated by comparison with a standard curve fit using a four parameter logistic regression model conducted in a GraphPad Prism software version 7.03.

### Statistical analyses

Data analysis was conducted using STATA version 13.1 (College Station, Texas). The laboratory data were merged with archived data from the KBC participant’ databases for analysis. Sample PRNT titres were logarithmically transformed (base 2) for all statistical analyses.

Correlation analyses between PRNT antibodies (log _2_PRNT) and IgG ELISA (log _10_) antibodies was done using a Pearson correlation test. Agreement between PRNT and ELISA was done using Bland-Altman plots ^23^. For each serum sample, the difference between PRNT and ELISA result was plotted against the averages of both assays. During Bland-Altman analysis, concentrations for RSV IgG IBL ELISA and crude lysate RSV IgG ELISA were log transformed to (base 2) and the data tested for normality. Modified Bland-Altman plots were used to test the effect of age on agreement between ELISA and PRNT. Regression analysis between ELISA and PRNT was used to estimate the 95% prediction intervals of PRNT antibodies by IgG ELISA method. The standard error (SE) of the predicted PRNT titre for an individual was estimated using the formula:

$$SE\;=\;\sqrt{\left(RMS\;\left(1\;+\;\frac{1}{n}\right)\right)},$$

Where, RMS is the residual mean squares and n is the number of observations. The predicted mean of PRNT titre by an ELISA method was calculated using the formula:

y=mx+c,

Where y is the predicted PRNT titre, *m* is the regression coefficient, *x* is the RSV antibody concentration by ELISA measurement and *c* is the y intercept where *x*=0. The upper and lower 95% prediction intervals were calculated using the formula: Upper/Lower 95% prediction limit = *(mx+c)* +/- 1.96SE, where SE is the standard error (see full methods here).

## Results

### Baseline characteristics

Archived sera from 100 cohort participants were selected. However, for 1 participant, the samples were not sufficient to screen for ELISA IgG antibodies after screening for PRNT titres. A total of 229 samples (99 cord samples, 87 three-month samples and 43 six-month samples) from the 99 participants were therefore available with data for the 3 serological assays. The mean gestational age at birth was 38.5 weeks (95% CI 37.6 – 39.3), while the mean birth weight was 2.9 kilograms (95% CI 2.7 – 3.0). The mean concentration of log _2_PRNT titres were: cord blood samples, 10.6 log _2_PRNT (95% Confidence interval, CI; 10.3– 10.8); 3 months of age samples; 8.3 log _2_PRNT (95% CI 8.1 – 8.6); and 6 months and over 7.6 log _2_PRNT (95% CI 7.1 – 8.1). The highest PRNT titre observed among these participants was 12.9 log _2_PRNT while the lowest was 5.1 log _2_PRNT.

### QA results

The mean PRNT titre results of a panel of 5 reference samples from BEI Resources tested in our laboratory were within range of the PRNT titre results as tested in BEI in 2011. These results are shown in Table 1.

**Table 1.** Neutralisation test results by Kenya Medical Research Institute KEMRI-Wellcome Trust Research Programme (KEMRI-WTRP) and BEI laboratories of samples from BEI Resources respiratory syncytial virus (RSV) Reference panel catalogue #NR-32832 which contains 5 different pooled human polyclonal antisera to RSV.

Reference sample	Expected titer range provided by BEI in 2011	KEMRI-WTRP
	in 2011	Mean log _2_ neut titer
BEI NR-4020 Wyeth lot 6594	8.79 (±6.48)	8.93 (8.61-9.20)
BEI NR-4021 Wyeth lot 6937 - high	10.88 (±9.75)	10.29 (9.91-10.60)
BEI NR-4022 Wyeth lot 6938 - medium	7.81 (±5.81)	8.75 (8.12-9.19)
BEI NR-4023 Wyeth lot 6939 - low	8.28 (±2.81)	8.73 (8.25-9.08)
BEI NR-21973 CBER reference Ig lot RSV-1	7.56 (±5.21)	8.59 (8.09-8.96)
Palivizumab (Synagis) 100 mg/ml		(Assayed a 1% solution of stock Ab) 10.59 (10.43-10.73) Undiluted = 17.23

### Comparison of ELISA with PRNT

**Figure 1.**
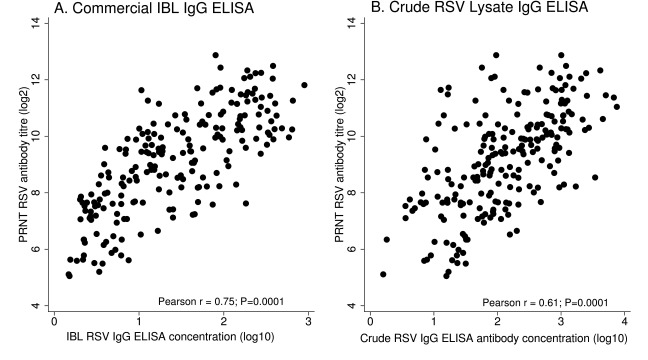
Scatter plots showing correlation between: ( **a**) Commercial IBL respiratory syncytial virus (RSV) IgG ELISA and plaque reduction neutralisation test (PRNT) RSV antibody titres ( **b**) Crude RSV lysate IgG ELISA antibody and PRNT RSV antibody titres. The spearman correlation coefficient (r) is shown. ELISA IgG antibodies are presented using base10 log scale, while PRNT RSV antibodies are presented using base 2 log scale.

A Pearson correlation test to determine the strength of association between ELISA antibodies and neutralizing antibody ( Figure 1), showed a strong positive correlation between PRNT titres and IBL ELISA antibody (r=0.75), and moderate positive correlation between PRNT titres and crude ELISA antibody (r=0.61). The two correlations were each statistically significant (P=0.0001).

**Figure 2.**
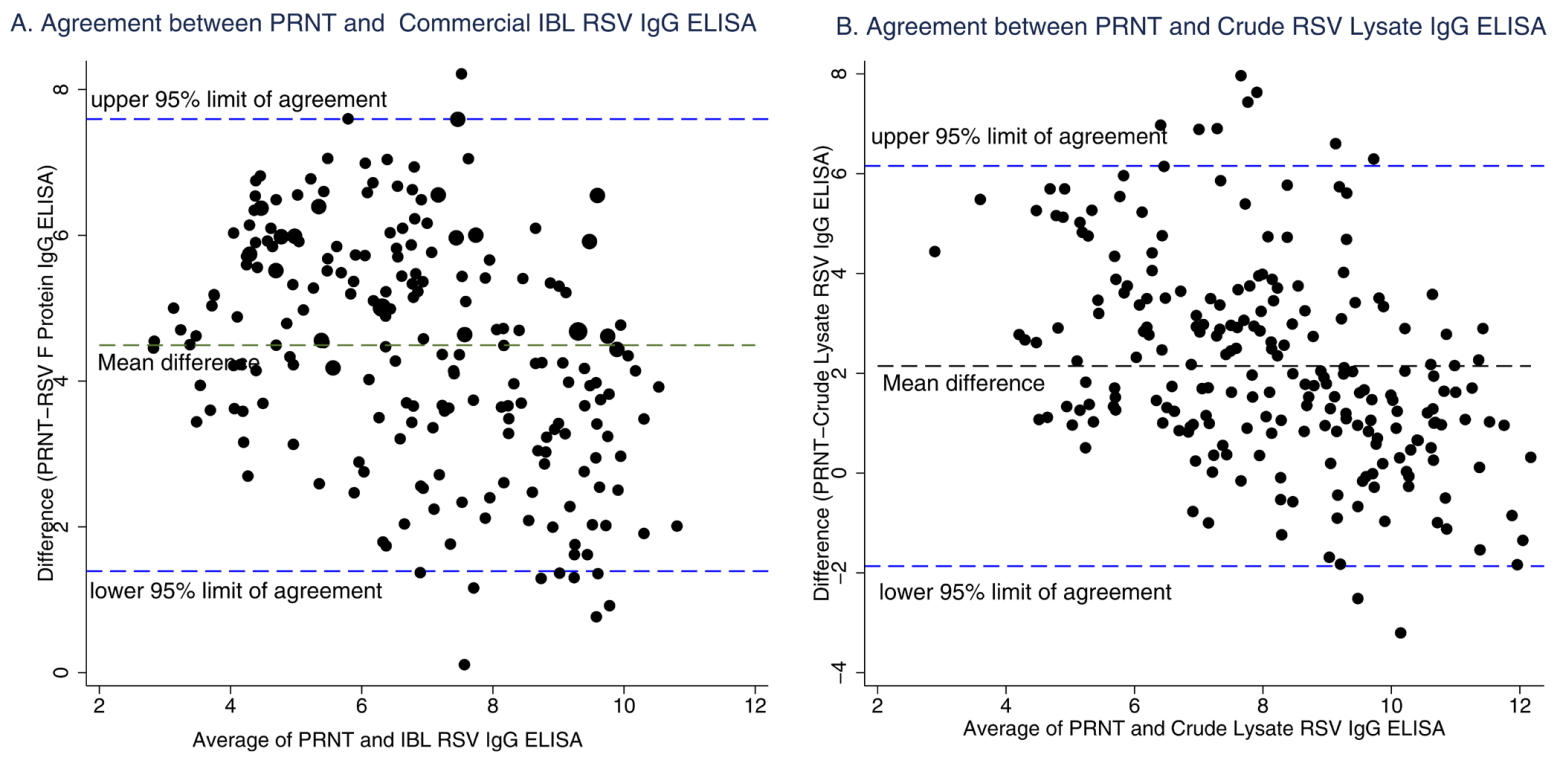
Bland Altman plots showing agreement between: ( **a**) Commercial IBL respiratory syncytial virus (RSV) IgG ELISA and plaque reduction neutralisation test (PRNT) RSV antibody titres ( **b**) Crude RSV lysate IgG ELISA antibody and PRNT RSV antibody titres. The 95% limits of agreement, mean difference and averages are also shown for each graph. PRNT and ELISA IgG antibodies are presented using base2 log scale.

The distribution of log transformed RSV antibody concentrations were approximately log- normal for each ELISA dataset and for the PRNT data. Bland-Altman plots were drawn to assess the agreement between PRNT and ELISA in quantifying a high or low RSV specific antibody level from a sample ( Figure 2). In this analysis, 95% of samples were observed to fall within the 95% level of agreement between PRNT and IBL ELISA and 96% of samples between PRNT and crude ELISA, respectively. The Bland-Altman plots showed a mean difference of 4.5 log _2_ PRNT (95% limits 1.3-7.6) between PRNT and IBL ELISA and 2.1 log _2_ PRNT (95% limits -1.9-6.2) between PRNT and crude ELISA. Crude RSV lysate IgG ELISA showed a slightly wider 95% limit of agreement (-1.866, 6.157) compared to IBL RSV IgG ELISA (1.392, 7.595).

**Figure 3.**
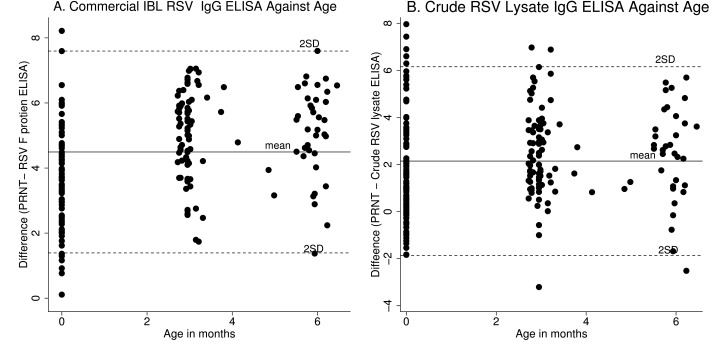
Modified Bland Altman scatter plot showing the effect of age on agreement between plaque reduction neutralisation test (PRNT) and ELISA assays. Panel **A** is a graph for difference between PRNT and Commercial IBL respiratory syncytial virus (RSV) IgG ELISA against age and panel **B** is a graph for difference between PRNT and Crude RSV lysate IgG ELISA against age. The short dashed gray lines represent the 95% limits of agreement, solid gray line represent the mean difference of agreement for each graph. PRNT and ELISA IgG antibodies are presented using base2 log scale.

Modified Bland-Altman plots ( Figure 3) show the difference in value of the ELISA and PRNT results plotted against age in months (i.e. time since birth of collection of the samples). Age did not have any appreciable effect on the agreement between PRNT and either of the ELISA result.

**Figure 4.**
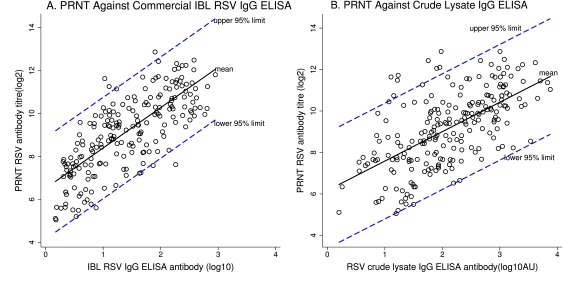
Scatter plots and regression analysis showing respiratory syncytial virus (RSV) specific antibody predictions between: ( **a**) IBL RSV IgG ELISA and plaque reduction neutralisation test (PRNT) RSV antibody titres ( **b**) Crude RSV lysate IgG ELISA antibody and PRNT RSV antibody titres. The 95% prediction intervals are shown for each graph by blue dashed lines, while the predicted mean for PRNT measurements by ELISA are shown using continuous black line. Black hollow circles represent individual antibody levels. ELISA IgG antibodies are presented using base10 log scale, while PRNT RSV antibodies are presented using base 2 log scale.

Further assessment using regression analysis ( Figure 4), estimated the standard error for predicting the mean PRNT titres by ELISA method to be 1.204 and 1.424 for IBL ELISA and crude ELISA, respectively. All samples with low concentrations of RSV specific antibody were within the 95% prediction limit for crude RSV lysate IgG ELISA ( Figure 4b). The predicted PRNT values by IBL ELISA were given as (1.880 *x* + 6.517), while the predicted PRNT values by crude ELISA were given as (1.419 *x* +6.164). The 95% prediction limits for PRNT antibodies were slightly higher for crude ELISA +/- 2.79 log _2_PRNT compared to IBL ELISA +/-2.36 log _2_PRNT.

## Discussion

To overcome the challenges experienced with PRNT as a technique for quantifying protective immunity correlates to RSV in large vaccine studies, we explored how well ELISA and PRNT methods agree in detecting levels of RSV specific antibodies, and in addition, investigated how accurate an ELISA method can predict the PRNT measurements and if it could be considered a suitable replacement for PRNT.

We found moderate to good correlations between each ELISA (IBL (r =0.75) and crude (r=0.61)) and the reference method for neutralizing antibodies, PRNT. The stronger correlation in IBL ELISA could be explained by the fact that, both PRNT and IBL ELISA methods were designed to target specific antibodies against the RSV F and G protein. This is because, neutralising antibodies are presumed to dominate within the RSV F protein also commonly known as the key target for neutralizing antibodies ^24, 25^. Consequently, there is increasing interest to use the RSV F protein as the maternal RSV vaccine candidate antigen ^7^ **.** The moderate correlation with crude ELISA is likely caused by the wide range of antibodies against multiple RSV antigens the assay targets. This might have possibly led to detection of additional antibodies specific to other RSV proteins that are missed by both IBL ELISA and PRNT assay.

We identified few studies that show correlation between ELISA and neutralization assay ^26– 29^. Similar findings to our study were previously reported by Welch *et al*, who suggested that ELISA measure all antibody types and do not discriminate the neutralizing antibodies as measured by functional assays, thus cannot be relied upon to predict the neutralizing activity of the sera ^29^. Due to the moderate correlation observed in our study between PRNT and crude RSV lysate IgG ELISA, we suggest that, careful consideration should be made on the choice of ELISA based assays as a surrogate for a neutralization assay in epidemiological studies.

The Bland-Altman plots demonstrated that 96% of all samples fell into the 95% limits of agreement between PRNT and crude ELISA. However, the crude ELISA had a slightly wider limit of agreement with PRNT compared to IBL ELISA. The PRNT values were also consistently higher than for ELISA. The wider limit of agreement indicates that the crude RSV lysate IgG ELISA has a higher variability with PRNT compared to IBL ELISA. This again, raises the issue of specificity and affinity of binding for RSV specific IgG antibodies measured using the crude ELISA method. Very few studies have used Bland-Altman plots to assess agreement between two serological methods ^29– 32^. One study which evaluated two commercially available ELISAs and one in-house reference laboratory ELISA for the determination of human anti-rabies virus antibodies ^29^, suggested that the results from the Bland-Altman plot analysis can only offer an insight into the availability of alternative, less complex method to monitor antibody titres during vaccine studies.

We also demonstrated that age did not have an appreciable effect on the agreement between PRNT and ELISA methods in detection of RSV specific antibodies. We performed this analysis because poor correlation between functional antibody and antibody concentration is thought to be influenced by age ^33^. For instance, samples from infants and the elderly are likely to show a poor correlation between PRNT and ELISA usually because their receptors don’t activate appropriate T or B cell responses, although the antibodies bind to antigen ^33^. However, in this study, both methods showed agreement in the level of detected antibody for an individual regardless of age.

In this study, we could predict PRNT titres using ELISA with moderate accuracy. Using regression analysis, the IBL ELISA method predicted the mean PRNT titre at 95% prediction interval within a width of 4.8log _2_ PRNT, while, crude RSV lysate IgG ELISA predicted the mean PRNT titre within a width of 5.6 log _2_ PRNT units. This implies that, the PRNT titre of any given serum sample if estimated using an ELISA IgG antibody concentration measured directly by the ELISA method would fall within 2.4-2.8 fold higher or lower than the true value. In a perfect regression, if RSV antibody concentrations were measured by ELISA, the corresponding PRNT values would be estimated using (1.880 *x* + 6.517) and (1.419 *x* +6.164) for IBL ELISA and crude ELISA respectively. With, *x* being the antibody concentration measured directly by the respective ELISA method. However, the high variability makes direct estimation of PRNT values by the above formula difficult. For instance, the 95% upper limit of detecting mean cord PRNT titre of 10.6 log _2_PRNT by crude ELISA would be 13.4log _2_PRNT, which is above the highest value (12.9log _2_PRNT) the PRNT assay detected in this study. The combined analysis approach we have applied on the data is unique and we think, this could better guide the choice of a suitable serological technique for use in place of a PRNT assay for large vaccine studies; and especially if the units of measurements between the two methods are different.

The main limitation we highlight in this study is that the PRNT assay we are using is difficult to standardize, which complicates comparison of results between laboratories. However, during the time this study was conducted there was no RSV standard available. Nevertheless, we tried to mitigate this limitation by using a panel of reference serum samples from BEI resources to monitor and minimise assay variability and to make sure results of the test samples obtained are acceptable. Another limitation is the systematic bias observed with analysis using Bland-Altman plots. This was due to the differences in metrics used to measure RSV specific antibodies by ELISA and PRNT. The RSV specific antibodies in ELISA are measured as a concentration (log10) while in PRNT assay are measured as titres (log2). The Bland-Altman plots show a trend if analysis is done using the respective metrics of the serological assays, thus difficulty in estimating the mean difference. The plots in Bland-Altman analysis should demonstrate that the two methods are consistent in what they are measuring in the same metrics. We tried to minimise this by transforming ELISA values to log base 2 during Bland-Altman plot analysis.

## Conclusions

Both ELISA methods show a moderate-good correlation with PRNT in measuring RSV specific antibodies. However, a commercial anti RSV based ELISA antibody assay which has less variability and has a high positive correlation with PRNT assay in detecting RSV specific antibody responses, does not provide an accurate prediction of PRNT antibody values. The true PRNT value would lie 2.4 fold higher or 2.4 fold lower if a commercial RSV IgG IBL ELISA is used in place of a PRNT assay. Our results suggest that, should PRNT prove unsuitable as a Gold standard for quantifying RSV specific antibodies, the utility of an ELISA method in vaccine studies should be assessed independent of a PRNT assay.

## Data availability

### Underlying data

The raw data is stored under restricted access and available from the authors upon request through submission of a request form http://kemri-wellcome.org/aboutus/#ChildVerticalTab_15 for consideration by our Data Governance Committee ( dgc@kemri-wellcome.org).

Harvard Dataverse: Replication Data for: Agreement between ELISA and Plaque Reduction Neutralisation Assay in Detection of Respiratory Syncytial Virus Specific Antibodies in a Birth Cohort from Kilifi, Coastal Kenya. https://doi.org/10.7910/DVN/K3LS7M ^34^

This project contains the following underlying data:

- NEUT ELISA Analysis script.do
- NEUT ELISA data codebook.pdf
- NEUT ELISA data file.tab
- NEUT ELISA_Data Readme txt.txt

Data are available under Creative Commons Attribution 4.0 International (CC BY 4.0) Licence

## References

[1] Lee MS Walker RE Mendelman PM: Medical burden of respiratory syncytial virus and parainfluenza virus type 3 infection among US children. Implications for design of vaccine trials. *Hum Vaccin.* 2005;1(1):6–11. 10.4161/hv.1.1.1424 17038832

[2] Cooney MK Fox JP Hall CE: The Seattle Virus Watch. VI. Observations of infections with and illness due to parainfluenza, mumps and respiratory syncytial viruses and *Mycoplasma pneumoniae*. *Am J Epidemiol.* 1975;101(6):532–551. 10.1093/oxfordjournals.aje.a112125 168766

[3] Noyola DE Arteaga-Dominguez G: Contribution of respiratory syncytial virus, influenza and parainfluenza viruses to acute respiratory infections in San Luis Potosi, Mexico. *Pediatr Infect Dis J.* 2005;24(12):1049–1052. 10.1097/01.inf.0000190026.58557.93 16371864

[4] Chanock R Chambon L Chang W: WHO respiratory disease survey in children: a serological study. *Bull World Health Organ.* 1967;37(3):363–369. 5301380 PMC2554267

[5] Nair H Nokes DJ Gessner BD: Global burden of acute lower respiratory infections due to respiratory syncytial virus in young children: a systematic review and meta-analysis. *Lancet.* 2010;375(9725):1545–55. 10.1016/S0140-6736(10)60206-1 20399493 PMC2864404

[6] Nokes DJ Ngama MJ Bett A: Incidence and severity of respiratory syncytial virus pneumonia in rural Kenyan children identified through hospital surveillance. *Clin Infect Dis.* 2009;49(9):1341–1349. 10.1086/606055 19788358 PMC2762474

[7] Raghunandan R Lu H Zhou B: An insect cell derived respiratory syncytial virus (RSV) F nanoparticle vaccine induces antigenic site II antibodies and protects against RSV challenge in cotton rats by active and passive immunization. *Vaccine.* 2014;32(48):6485–6492. 10.1016/j.vaccine.2014.09.030 25269094 PMC7172787

[8] Piedra PA Jewell AM Cron SG: Correlates of immunity to respiratory syncytial virus (RSV) associated-hospitalization: establishment of minimum protective threshold levels of serum neutralizing antibodies. *Vaccine.* 2003;21(24):3479–3482. 10.1016/S0264-410X(03)00355-4 12850364

[9] Sande CJ Mutunga MN Medley GF: Group- and genotype-specific neutralizing antibody responses against respiratory syncytial virus in infants and young children with severe pneumonia. *J Infect Dis.* 2013;207(3):489–492. 10.1093/infdis/jis700 23175761 PMC3541697

[10] Nyiro JU Sande C Mutunga M: Quantifying maternally derived respiratory syncytial virus specific neutralising antibodies in a birth cohort from coastal Kenya. *Vaccine.* 2015;33(15):1797–1801. 10.1016/j.vaccine.2015.02.039 25725445 PMC4376380

[11] Suara R Piedra PA Glezen WP: Prevalence of neutralizing antibody to respiratory syncytial virus in sera from mothers and newborns residing in the Gambia and in The United States. *Clin Diagn Lab Immunol.* 1996;3(4):477–479. 8807217 10.1128/cdli.3.4.477-479.1996 PMC170373

[12] Glezen WP Paredes A Allison JE: Risk of respiratory syncytial virus infection for infants from low-income families in relationship to age, sex, ethnic group, and maternal antibody level. *J Pediatr.* 1981;98(5):708–715. 10.1016/S0022-3476(81)80829-3 7229749

[13] Roca A Abacassamo F Loscertales MP: Prevalence of respiratory syncytial virus IgG antibodies in infants living in a rural area of Mozambique. *J Med Virol.* 2002;67(4):616–623. 10.1002/jmv.10148 12116014

[14] Piedra PA Hause AM Aideyan L: Respiratory Syncytial Virus (RSV): Neutralizing Antibody, a Correlate of Immune Protection. *Methods Mol Biol.* 2016;1442:77–91. 10.1007/978-1-4939-3687-8_7 27464689

[15] Pastrana DV Buck CB Pang YY: Reactivity of human sera in a sensitive, high-throughput pseudovirus-based papillomavirus neutralization assay for HPV16 and HPV18. *Virology.* 2004;321(2):205–216. 10.1016/j.virol.2003.12.027 15051381

[16] Stensballe LG Simonsen JB Thomsen SF: The causal direction in the association between respiratory syncytial virus hospitalization and asthma. *J Allergy Clin Immunol.* 2009;123(1):131–137.e1. 10.1016/j.jaci.2008.10.042 19130934

[17] Hosken N Plikaytis B Trujillo C: A multi-laboratory study of diverse RSV neutralization assays indicates feasibility for harmonization with an international standard. *Vaccine.* 2017;35(23):3082–3088. 10.1016/j.vaccine.2017.04.053 28476625 PMC5439532

[18] Nokes DJ Okiro EA Ngama M: Respiratory syncytial virus epidemiology in a birth cohort from Kilifi district, Kenya: infection during the first year of life. *J Infect Dis.* 2004;190(10):1828–1832. 10.1086/425040 15499540

[19] English M Muhoro A Aluda M: Outcome of delivery and cause-specific mortality and severe morbidity in early infancy: a Kenyan District Hospital birth cohort. *Am J Trop Med Hyg.* 2003;69(2):228–232. 10.4269/ajtmh.2003.69.228 13677381

[20] Scott JA Bauni E Moisi JC: Profile: The Kilifi Health and Demographic Surveillance System (KHDSS). *Int J Epidemiol.* 2012;41(3):650–657. 10.1093/ije/dys062 22544844 PMC3396317

[21] Ochola R Sande C Fegan G: The level and duration of RSV-specific maternal IgG in infants in Kilifi Kenya. *PLoS One.* 2009;4(12):e8088. 10.1371/journal.pone.0008088 19956576 PMC2779853

[22] Nyiro JU Kombe IK Sande CJ: Defining the vaccination window for respiratory syncytial virus (RSV) using age-seroprevalence data for children in Kilifi, Kenya. *PLoS One.* 2017;12(5):e0177803. 10.1371/journal.pone.0177803 28531224 PMC5439681

[23] Bland JM Altman DG: Applying the right statistics: analyses of measurement studies. *Ultrasound Obstet Gynecol.* 2003;22(1):85–93. 10.1002/uog.122 12858311

[24] McLellan JS Yang Y Graham BS: Structure of respiratory syncytial virus fusion glycoprotein in the postfusion conformation reveals preservation of neutralizing epitopes. *J Virol.* 2011;85(15):7788–7796. 10.1128/JVI.00555-11 21613394 PMC3147929

[25] Taleb SA Al Thani AA Al Ansari K: Human respiratory syncytial virus: pathogenesis, immune responses, and current vaccine approaches. *Eur J Clin Microbiol Infect Dis.* 2018;37(10):1817–1827. 10.1007/s10096-018-3289-4 29876771

[26] Westenbrink F Brinkhof JM Straver PJ: Comparison of a newly developed enzyme-linked immunosorbent assay with complement fixation and neutralisation tests for serology of bovine respiratory syncytial virus infections. *Res Vet Sci.* 1985;38(3):334–340. 10.1016/S0034-5288(18)31805-8 4012035

[27] Rabenau HF Marianov B Wicker S: Comparison of the neutralizing and ELISA antibody titres to measles virus in human sera and in gamma globulin preparations. *Med Microbiol Immunol.* 2007;196(3):151–155. 10.1007/s00430-007-0037-2 17308917

[28] Zhao H Lin ZJ Huang SJ: Correlation between ELISA and pseudovirion-based neutralisation assay for detecting antibodies against human papillomavirus acquired by natural infection or by vaccination. *Hum Vaccin Immunother.* 2014;10(3):740–746. 10.4161/hv.27619 24384608 PMC4130265

[29] Welch RJ Anderson BL Litwin CM: An evaluation of two commercially available ELISAs and one in-house reference laboratory ELISA for the determination of human anti-rabies virus antibodies. *J Med Microbiol.* 2009;58(Pt 6):806–810. 10.1099/jmm.0.006064-0 19429758

[30] Welch RJ Litwin CM: Evaluation of two immunoblot assays and a Western blot assay for the detection of antisyphilis immunoglobulin g antibodies. *Clin Vaccine Immunol.* 2010;17(1):183–184. 10.1128/CVI.00279-09 19940043 PMC2812094

[31] Weissbach FH Hirsch HH: Comparison of Two Commercial Tick-Borne Encephalitis Virus IgG Enzyme-Linked Immunosorbent Assays. *Clin Vaccine Immunol.* 2015;22(7):754–760. 10.1128/CVI.00096-15 25924768 PMC4478524

[32] Parreiras PM Sirota LA Wagner LD: Comparability of ELISA and toxin neutralization to measure immunogenicity of Protective Antigen in mice, as part of a potency test for anthrax vaccines. *Vaccine.* 2009;27(33):4537–4542. 10.1016/j.vaccine.2009.05.045 19501205

[33] Metcalf TU Cubas RA Ghneim K: Global analyses revealed age-related alterations in innate immune responses after stimulation of pathogen recognition receptors. *Aging Cell.* 2015;14(3):421–432. 10.1111/acel.12320 25728020 PMC4406671

[34] Nyiro JU Mwango L Nokes DJ: Replication Data for: Agreement between ELISA and Plaque Reduction Neutralisation Assay in Detection of Respiratory Syncytial Virus Specific Antibodies in a Birth Cohort from Kilifi, Coastal Kenya". Harvard Dataverse, V1, UNF:6:cXM/Pb4otN6UNHbP6wuvDA== [fileUNF].2019 10.7910/DVN/K3LS7M PMC6426078 30906883

