# Supplementary material for: Agreement between ELISA and plaque reduction neutralisation assay in Detection of respiratory syncytial virus specific antibodies in a birth Cohort from Kilifi, coastal Kenya
Source: Wellcome Open Res. 2019 Feb 18;4:33. [Version 1] doi: 10.12688/wellcomeopenres.15108.1 (PMC6426078; doi:10.12688/wellcomeopenres.15108.1)
Supplement: Peer review history

# Peer review: Reviewer response for version 1

**DOI:** 10.21956/wellcomeopenres.16484.r34954

**Published:** 2019-03-21

**Authors:** Pedro A. Piedra

**Affiliations:** 1 Department of Molecular Virology and Microbiology, Baylor College of Medicine, Houston, TX, USA

**Overall comment**.

 The major objective of this manuscript was to determine if at least one of the two in house RSV ELISA assays could predict with accuracy the neutralizing antibody titers generated by a plaque reduction neutralization test (PRNT). Although the methodology used to make the comparisons and analytical approach used to make the prediction are sound, the basic assumption is flawed when using crude or poorly described viral antigens in the ELISA assays. The neutralization assay primarily measures neutralizing antibodies directed to site-specific sites on the F protein while the ELISA assay using viral antigens will measure binding antibodies direct to the surface glycoproteins (F, G & SH) as well as the internal proteins (N, P, M, M2). Although good correlations are normally seen between ELISA and neutralization assays, it is entirely a different proposition to predict with accuracy neutralizing antibody titers when using viral antigens in an ELISA assay. Even site-specific competitive antibody assays to the F protein are not able to predict with accuracy neutralizing antibody tiers measured by neutralization assays because the immune response of individuals vary to the antigenic sites (Ø, II, IV, III, V) of the F protein even though they may have comparable neutralizing antibody activity. The lack of precision was evident in the results with the potential to have up to a 16 to 32-fold swing in the neutralizing antibody titer prediction using the ELISA assays.  

**Other comments**.

 In the manuscript PRNT is described as the preferred technique to determine immune correlates of protection. It is important to point out that the PRNT is one of many neutralization assays that have been developed for measuring neutralizing antibody activity against RSV. There are two major classes of neutralization assays; the PRNT and the microneutralization assay. There are many assay variations depending on the cell line, the virus inoculum, the signal or read out used to measure neutralizing activity, the duration of the assay, etc. Many of the PRNT references used in the manuscript actually refer to the classical microneutralization assay. It is also important to note that these neutralization antibody assays, in particular those without complement, primarily detect neutralizing antibody directed to the F protein and not the G protein.

 Neutralization assays are considered the best current method to evaluate the protective potential (neutralizing antibodies) induced by RSV infection or candidate vaccines. We now have an international standard (National Institute of Biological Standards and Control-NIBSC) against RSV/A (also being developed for RSV/B) that can be used to adjust neutralizing antibody titers generated by different neutralization assays into IU. This will greatly facilitate the comparisons of neutralizing antibody activity measured by different assays and induced by different candidate vaccines. The introduction section should be revised to reflect these up-dates.

**Introduction**.

 The Fusion (F) protein in Novavax F nanoparticle vaccine is not a true post-fusion F protein vaccine. It is consistent with a prefusogenic form that is able to generate site-specific antibody responses to both the pre-fusion and post-fusion F conformations ^1^.

**Method**.

 What was the collection tube used to process plasma? Plasma collected in heparin coated tubes will inaccurately measure plasma samples with low neutralizing antibody activity to RSV because heparin containing plasma will act as an antiviral agent. The effect is to inhibit virus replication when in reality there is no neutralizing antibody activity. This is unlikely to have an effect with plasma samples with moderate to high levels of neutralizing antibody titers against because the effect is diluted out with increasing two-fold dilutions.

 For the ELISA assays it is unclear what the dilution scheme was for generating the ELISA units. The results are presented in log10, which suggest a dilution scheme of 10-fold. Please clarify the dilution scheme for the two ELISA assays. Also for all the assays please describe the range of the antibody titer and arbitrary unit for the assays. It would also be helpful to know the coefficient of variation for these assays at the lower limit of detection, dynamic range and upper limit of detection.

 It is unclear the viral antigen(s) included in the IBL ELISA assay. The information is not provided in the IBL package insert.

**Results**.

 It is a misnomer to call it an RSV-F ELISA. Please see Y axis for Figures 2 and 3.

 Figures 2 & 3, Y axis is the difference (PRNT – RSV F protein ELISA) in log _2_?

 What was the correlation between the two ELISA assays, and did one ELISA assay predict with good accuracy the AU of the other ELISA assay. That would serve as an internal validation of the statistical method used to predict neutralizing antibody titer with ELISA bind antibody data.

**Discussion**.

 Page 8, 7 ^th^ paragraph, “predict with moderate accuracy”. Having a width of 4.8 log to 5.6 log _2_ is huge and should be considered imprecise.

 In my opinion, the main limitation is not the standardization of the PRNT but rather the assumption that accurate predictions can be made when using ELISA assays with poorly characterized viral antigens. A second major limitation is the use of immunological assays that do not appear to have been qualified. Information on the reproducibility, dynamic range, lower and upper limit of detection were not provided for these assays.

I have read this submission. I believe that I have an appropriate level of expertise to confirm that it is of an acceptable scientific standard, however I have significant reservations, as outlined above.

## References

[1] GilbertBEPatelNLuHLiuYGuebre-XabierMPiedraPAGlennGEllingsworthLSmithG: Respiratory syncytial virus fusion nanoparticle vaccine immune responses target multiple neutralizing epitopes that contribute to protection against wild-type and palivizumab-resistant mutant virus challenge. *Vaccine*.2018;36(52) : 10.1016/j.vaccine.2018.10.073 8069-8078 10.1016/j.vaccine.2018.10.073 30389195

# Peer review: Reviewer response for version 1

**DOI:** 10.21956/wellcomeopenres.16484.r34920

**Published:** 2019-03-19

**Authors:** Helen Y. Chu

**Affiliations:** 1 Department of Medicine, University of Washington, Seattle, WA, USA

This is an important study which addresses the question of whether RSV ELISAs can replace neutralizing antibody assays for use in vaccine trials. This study analyzes the agreement between and accuracy of measuring RSV specific antibodies using ELISA w/ crude RSV lysate and RSV IgG ELISA in comparison with the current “gold standard” method for measuring RSV specific antibodies, a plaque reduction neutralization test (PRNT). In previous studies, ELISA and PRNT have not been shown to correlate well, due to non-neutralizing antibodies. 

 Methodologically, the study is sound. Comparison was made between the PRNT, utilizing a method that has been used by multiple labs. Results from a standardized set of reference sera are included, showing performance of the test within range. Comparison was made against a commercial and in-house ELISA. Samples tested included sera samples from the cord blood of 99 participants and subsequent 3 and 6-month samples from a fraction of these participants. Strength of association was calculated with a correlation test and agreement between PRNT and ELISA were examined on Bland-Altman plots.

 Results demonstrated a relatively high correlation was seen between the crude RSV Lysate ELISA and PRNT, and a more moderate correlation was observed between the RSV IgG ELISA and PRNT. Overall, this is an important study in the setting of multiple new vaccine and antibody prophylaxis candidates in clinical trials, for which it would be immensely helpful to have a less expensive, less labor-intensive method of measuring antibody as a correlate of protection.

 Overall the commercial ELISA had higher correlation as compared to the ELISA using crude RSVA2 lysate, which as the authors point out, is likely due to targeting of F and G antigen in the commercial assay. Despite this higher correlation, the margin of error for the formula calculating the relationship between the ELISA and the PRNT is too high to be useful. This is a useful study, and reaffirms the use of RSV neutralizing antibody assays as the gold standard. One minor criticism, largely outside the scope of this study, is the consideration of other correlates of protection beyond neutralizing antibody as markers of protection against disease.

I have read this submission. I believe that I have an appropriate level of expertise to confirm that it is of an acceptable scientific standard.

# Peer review: Reviewer response for version 1

**DOI:** 10.21956/wellcomeopenres.16484.r34921

**Published:** 2019-03-06

**Authors:** Shabir Madhi

**Affiliations:** 1 University of Witwatersrand, Johannesburg, South Africa

This study evaluated the agreement between the RSV plaque reduction neutralisation assay (PRNT), a commercially available RSV IgG ELISA assay and an in-house RSV assay using crude RSV lysate as an antigen.

 General comments:

1. Overall, the methods and results section of the manuscript are well written and analysed. 
2. Although the study objective is clear, the rational for the study is more ambiguous. The main issue at hand in the context of vaccine development, is the need for an assay which can predict the efficacy of the vaccine –and ideally a functional assay or at least one that bridges with a functional assay. This would be important for bridging studies, in the absence of being able to undertake multiple large scale vaccine efficacy/effectiveness studies. Although the authors cite some studies reporting a positive association of PRNT antibody and risk/odds of disease, omitted from the background are other studies (including one from the same group)- which actually reported no association between PRNT and RSV LRTI. This calls into question the value of the current “gold standard”- i..e PRNT- on its own (over and above the issues with regard to standardisation and being labour intensive) in relation to whether or not it would be useful for establishing a correlate of protection.
  In the context of this study where the ELISA assays, as also noted by the authors, are not discriminatory in terms of measure of antibody to epitopes specifically targeted by most of the vaccines currently under clinical development (including the nanoparticle F-fusion vaccine), the likelihood of any predictive value of a relatively non-specific ELISA assay being of use to establish or impute a correlate of protection would be doubtful from the outset.
  These issue merit inclusion in the abstract, study rational and the discussion.
3. The measure of RSV antibody could have multiple purposes- including characterising sero-epidemiology, measuring vaccine immunogenicity or trying to establish a correlate of protection. Depending on for what purpose the assay is being used, would influence the minimum characteristics required of the assay. In the context of this study, although the ELISA assays were only moderately predictive of the PRNT antibody measure, this does not necessarily preclude the use thereof in sero-epidemiology studies. Likewise, even if there was a strong correlation between the ELISA and PRNT assays, in the absence of an established correlate of protection using the PRNT assay- that too would not be of much value in terms of being used for studies to impute vaccine effectiveness in different settings.

Specific comments

1. Pg 3 end para 2- need to clarify that the standardised assays which are required need to be assays that are predictive of vaccine protection. Also, clarify that the main purpose of such assays are to undertake “bridging immunogenicity studies” to avoid multiple vaccine efficacy/effectiveness studies. Such assays will need to be tested for predicting vaccine efficacy often within a clinical trial and the assays could differ based on the construct of the vaccine (i.e differences in specific epitopes targeted).
2. Pg 3 para 3- mention and reference studies that have shown no association between PRNT and LRTI.
3. Pg 3 para 4- clarify that the needs of an assay for sero-epi studies may well differ to those required to establish a correlate of protection.
4. Pg 3 para 5- what would the need for a serological assay be in a “large vaccine efficacy trial” which will provide the most definitive answer on vaccine efficacy?
5. Under lab methods- clarify where the lab assays were done.
6. Pg 8 para 3- neutralizing antibodies are also elicited by immune responses to the G and M protein. This might well explain some of the conflicting findings on the association of PRNT and risk for RSV LRTI.
7. Pg 8 para 4- the last sentence needs to be elaborated upon, including what the possibility for epitope specific antibody read-outs that might well differ between vaccine constructs.  

I have read this submission. I believe that I have an appropriate level of expertise to confirm that it is of an acceptable scientific standard, however I have significant reservations, as outlined above.

# Peer review: Reviewer response for version 1

**DOI:** 10.21956/wellcomeopenres.16484.r34922

**Published:** 2019-03-05

**Authors:** Ting Shi

**Affiliations:** 1 University of Edinburgh, Edinburgh, UK

This study investigates how well ELISA and PRNT methods agree in detecting levels of of RSV specific antibodies (also accuracy) and whether an ELISA method can be an appropriate replacement for PRNT. Generally it is well written and I only have minor comments listed as follows:

1. Can you please justify the sample size you selected for the purpose of this study?
2. You mentioned that age did not have a substantial effect on the agreement between PRNT and ELISA methods. As age advances, will you expect the similar finding?
3. What is your opinion of applying the similar ELISA methods quantifying RSV specific antibodies beyond young children? e.g. older adults?
4. Will different clinical specimen make an influence on the agreement / accuracy of ELISA methods?
5. What is the next step (research question) based on findings from this study?

I have read this submission. I believe that I have an appropriate level of expertise to confirm that it is of an acceptable scientific standard.
